# Plant membrane assays with cytokinin receptors underpin the unique role of free cytokinin bases as biologically active ligands

**DOI:** 10.1093/jxb/eru522

**Published:** 2015-01-21

**Authors:** Sergey N. Lomin, Dmitry M. Krivosheev, Mikhail Yu. Steklov, Dmitry V. Arkhipov, Dmitry I. Osolodkin, Thomas Schmülling, Georgy A. Romanov

**Affiliations:** ^1^Institute of Plant Physiology, Russian Academy of Sciences, Botanicheskaya 35, 127276 Moscow, Russia; ^2^Department of Chemistry, Lomonosov Moscow State University, Leninskie Gory 1/3, 119991 Moscow, Russia; ^3^Institute of Biology/Applied Genetics, Dahlem Centre of Plant Sciences, Freie Universität Berlin, Albrecht-Thaer-Weg 6, D-14195 Berlin, Germany; ^4^Belozersky Institute of Physico-Chemical Biology, Lomonosov Moscow State University, Leninskie Gory 1, 119992 Moscow, Russia

**Keywords:** *Arabidopsis thaliana*, cytokinin, cytokinin receptor, ligand specificity, pH sensor, plant assay system, receptor binding assay, *Zea mays.*

## Abstract

Cytokinin receptors play a key role in cytokinin-dependent processes regulating plant growth, development, and adaptation; therefore, the functional properties of these receptors are of great importance. Previously the properties of cytokinin receptors were investigated in heterologous assay systems using unicellular microorganisms, mainly bacteria, expressing receptor proteins. However, within microorganisms receptors reside in an alien environment that might distort the receptor properties. Therefore, a new assay system has been developed allowing studies of individual receptors within plant membranes (i.e. closer to their natural environment). The main ligand-binding characteristics of receptors from *Arabidopsis* [AHK2, AHK3, and AHK4] and maize (ZmHK1) were refined in this new system, and the properties of full-length *Arabidopsis* receptor AHK2 were characterized for the first time. Ligand specificity profiles of receptors towards cytokinin bases were comparable with the profiles retrieved in bacterial assay systems. In contrast, cytokinin-9-ribosides displayed a strongly reduced affinity for receptors in the plant assay system, indicating that ribosides as the common transport form of cytokinins have no or very weak cytokinin activity. This underpins the central role of free bases as the sole biologically active cytokinin compounds. According to molecular modelling and docking studies, *N* ^9^-ribosylation alters the bonding pattern in cytokinin–receptor interaction and prevents β6–β7 loop movement important for tight hormone binding. A common feature of all receptors was a greatly reduced ligand binding at low (5.0–5.5) pH. The particularly high sensitivity of ZmHK1 to pH changes leads to the suggestion that some cytokinin receptors may play an additional role as pH sensors in the lumen of the endoplasmic reticulum.

## Highlight

Cytokinin receptors studied in a novel plant assay system recognize cytokinin ribosides poorly, unlike cytokinin bases. Molecular modelling explained this receptor feature. Some receptors were suggested to function as pH sensors.

## Introduction

Cytokinins are important plant hormones regulating numerous aspects of plant development and physiology (Sakakibara, 2006; Werner and Schmülling, 2009; Romanov, 2009; Hwang *et al.*, 2012; Kieber and Schaller, 2014). The cytokinin signal is perceived by membrane-spanning sensor histidine kinases, which feed into a multistep phosphorelay signal transduction system (for recent reviews, see Müller, 2011; Heyl *et al.*, 2012; Spíchal, 2012; Lomin *et al.*, 2012; Shi and Rashotte, 2012; El-Showk *et al.*, 2013; Steklov *et al.*, 2013). Upon the discovery of cytokinin receptors (Inoue *et al.*, 2001; Suzuki *et al.*, 2001) their functional properties were extensively studied (Yamada *et al.*, 2001; Spíchal *et al.*, 2004; Yonekura-Sakakibara *et al.*, 2004; Mok *et al.*, 2005; Romanov *et al.*, 2005, 2006; Lomin *et al.*, 2011; Stolz *et al.*, 2011; Kuderová *et al.*, 2015). Many important biochemical parameters of these receptors, including ligand specificity, lack of binding co-operativity, and pH, temperature, and salt dependences of hormone binding, were determined (Romanov *et al.*, 2005, 2006; Lomin and Romanov, 2008; Lomin *et al.*, 2011; Stolz *et al.*, 2011). The ligand-binding properties of individual cytokinin receptors have been studied so far in heterologous, mainly bacterial and occasionally yeast assay systems (Higuchi *et al.*, 2009; Romanov and Lomin, 2009; Mizuno and Yamashino, 2010; Spíchal, 2011). Such transgenic test systems, particularly those based on bacteria, have a number of advantages, namely the capacity to accumulate high amounts of foreign protein, the absence of specific enzymes for cytokinin modification and degradation, and the relative ease of practical work. However, bacterial membranes are known to differ from plant membranes in their lipid and protein composition as well as other parameters such as thickness or water content (Opekarová and Tanner, 2003; Pogozheva *et al.*, 2013; Grimard *et al.*, 2014). Therefore, in a heterologous system receptors reside in an alien membrane environment which might affect receptor properties. Such an environment may be playing a significant role particularly in view of recently uncovered consensus motifs in transmembrane helices of cytokinin receptors (Steklov *et al.*, 2013). Thus, to ascertain the genuine functional characteristics of cytokinin receptors, it would be important to study them in an environment as close as possible to their natural environment. To this end, a plant assay system was developed and its applicability was tested using a full receptor set from *Arabidopsis* (AHK2, AHK3, and AHK4, the last also known as CRE1 and WOL) and ZmHK1 from maize. Each of these receptors belongs to each of the three main evolutionary branches of these proteins (Pils and Heyl, 2009; Lomin *et al.*, 2012; Steklov *et al.*, 2013) and has distinct ligand specificities according to previous heterologous assays. In particular, AHK3 was shown to have the highest affinity for *trans*-zeatin (tZ), much lower affinity for isopentenyladenine (iP), and the lowest affinity for *cis*-zeatin (cZ) and *N* ^6^-benzyladenine (BA) (reviewed in Heyl *et al.*, 2012; Lomin *et al.*, 2012). AHK2 was previously studied in the form of a truncated protein only; the ligand specificity of the AHK2 sensor module was similar to that of AHK4, with the highest and similar affinities for iP and tZ followed by BA and cZ (Stolz *et al.*, 2011). The investigation of full-length AHK2 in the heterologous test system was not possible since it could hardly be expressed in *Escherichia coli*. Therefore, the need to find a way to study membrane receptors that are difficult to express in bacteria poses an additional challenge in this scientific area. ZmHK1, the maize orthologue of AHK4, was shown to have unique properties as it was distinguished by its strong preference for iP and BA while the affinity for tZ was much lower and close to the affinity for cZ.

All cytokinin receptors studied so far were shown to interact tightly with and respond to cytokinin ribosides (Heyl *et al.*, 2012), leading to the common notion that ribosides, similarly to free bases, are direct ligands for receptors. However, according to a more recent X-ray crystallography study, the cytokinin receptor AHK4 formed complexes with different cytokinin bases but not with tZ riboside (Hothorn *et al.*, 2011). Therefore, the question of whether or not cytokinin ribosides have genuine hormonal activity *in planta* still remains to be answered. This is biologically relevant as tZ riboside and iP riboside are the main transport form of cytokinins in the long-distance translocation via xylem and phloem, respectively (Sakakibara, 2006; Hirose *et al.*, 2008). The eventual necessity to form the free base from translocated cytokinin ribosides before it becomes an active hormone in target tissue evidently has important implications for the molecular processes required for this activation. Moreover, the concentrations of cytokinin ribosides found in plant tissue are often higher than those of the free base and are taken as a measure of the available active cytokinin. Clarifying the question of whether or not ribosides are biologically active would help to interpret the results of hormonal measurements correctly. In fact, the plant membrane assay system described here provided evidence that only the free cytokinin base has genuine hormonal activity. By means of a modelling approach, a molecular basis for the contrasting interaction of cytokinin bases and ribosides with the receptors was suggested. The plant assay system also enabled the influence of different parameters on receptor activity to be tested. In particular, a surprisingly strong pH dependence of ZmHK1 activity was revealed. The novel assay system is proposed as a useful tool for the analysis of the numerous plant membrane receptors within the plant environment.

## Materials and methods

### Plasmids and recombinant DNA techniques

Expression vectors pSTV28-AHK3 (Spíchal *et al.*, 2004; Miwa *et al.*, 2007) and pINIIIA3(ΔEH)-ZmHK1 (Yonekura-Sakakibara *et al.*, 2004) containing the coding sequences for the *AHK3* and *ZmHK1* receptor genes, respectively, were used in *E. coli* strain KMI001 K-12 [*rcsC::Kmr*, *wza::lacZ* (*cps-operon*), ∆*RcsC*] (Yamada *et al.*, 2001; Miwa *et al.*, 2007). For *Nicotiana benthamiana* transient transformation, the pB7FWG2-AHK3, pSPYCE-gAHK2, and pSPYNE-gAHK2 expression vectors (Wulfetange *et al.*, 2011) containing cDNA sequence for the *AHK3* and the genomic sequence of the *AHK2* gene, respectively, were used. To construct expression vector pB7FWG2-ZmHK1, the *AHK3* sequence was removed from pB7FWG2-AHK3 at the *Bcu*I/*Eco*RI sites. The *ZmHK1* coding region was amplified by PCR using the primers F (*ZmHK1/Bcu*I) 5′-GTGCG*ACTAGT*AAAATGGGGGGCAAGTA-3′ and R (*ZmHK1/Eco*RI) 5′-ATC*GAATTC*CCAACCTCTTGAGGTG AT-3′, and the vector pINIIIA3(ΔEH)-ZmHK1 as a template, and ligated into the vector pB7FWG2. Constructs based on the vector pB7FWG2 coded for receptors fused at their C-terminus to the fluorescent eGFP (Karimi *et al.*, 2007). The genomic *AHK4* sequence was amplified by PCR from bacterial artificial chromosome (BAC) clone T23K3 using the primers F (*AHK4/XbaI*) 5′-ACG*TCTAGA*ATGAGAAGAGATTTTGTG-3′ and R (*AHK4/Cfr9I*) 5′-AT*CCCGGG*CGACGAAGGTGAGAT-3′, and ligated into vectors pSPYNE and pSPYCE. All receptor genes were positioned under control of the 35S *Cauliflower mosaic virus* (CaMV) promoter. *Escherichia coli* transformation was accomplished by a simplified heat shock–calcium chloride method (Sambrook and Russell, 2001), and *Agrobacterium tumefaciens* transformation (strain GV3101) was carried out by the freeze–thaw shock method (Miller, 1987).

### E.coli spheroplast and membrane isolation

Isolation of *E. coli* spheroplasts (i.e. bacteria lacking the outer envelope) and membranes was performed as described previously (Romanov *et al.*, 2005; Lomin *et al.*, 2011).

### Transient expression of receptor genes in tobacco plants

The transient transformation of tobacco (*N. benthamiana*) leaves was carried out according to Sparkes *et al.* (2006). *Agrobacterium tumefaciens* carrying cytokinin receptor genes fused to reporter sequences, and the helper strain p19 (Voinnet *et al.*, 2003) were grown for 1–2 d at 28 °C in 1ml of LB medium as a pre-culture. Then 50ml of LB medium was inoculated by 0.5ml of pre-culture and incubated for 16h at 28 °C. Bacteria were pelleted for 5min at 10000 *g* at room temperature and resuspended in the infiltration solution (10mM MES-KOH, pH 5.7, 10mM MgCl_2_, 0.15mM acetosyringone), then pelleted again for 3min at 10 000 *g* and resuspended in 5ml of the infiltration solution. Tobacco plants at 5–6 weeks old were infiltrated with a mixture of telic (OD_600_ ~0.7) and p19 (OD_600_ ~1.0) agrobacterial strains, and the expression of receptor genes was checked after 4 d using a confocal microscope before leaves were processed further for microsome isolation.

### Plant membrane isolation

All manipulations were done at 4 °C. Tobacco leaves were homogenized in buffer containing 300mM sucrose, 100mM TRIS-HCl (pH 8.0), 10mM Na_2_-EDTA, 0.6% polyvinylpyrrolidone K30, 5mM K_2_S_2_O_5_, 5mM dithiothreitol (DTT), and 1mM phenylmethylsulphonyl fluoride (PMSF). The homogenate was filtered through Miracloth (Calbiochem, San Diego, CA, USA), and the filtrate was centrifuged for 10min at 10 000 *g*. Then the supernatant was centrifuged for 30min at 100 000 *g*. The microsome pellet was resuspended in 10mM phosphate-buffered saline (PBS; pH 7.4) or special media (see below) for pH dependence studies. After freezing, the microsome suspension can be stored at –70 °C.

### Hormone binding assays

Binding assays were performed at 0–4 °C and pH 7.4 except experiments aiming to study temperature or pH effects on hormone–receptor interaction. Highly labelled [2-^3^H]*trans*-zeatin ([^3^H]tZ, 851 GBq mmol^–1^, radiochemical purity >99%) was obtained from the Institute of Experimental Botany (Prague, Czech Republic). For one probe, 2.6 pmol [^3^H]tZ was used. An aliquot of 750 μl of spheroplast or membrane suspension (not less than 25 μg of protein in PBS) was mixed with 2.5 μl of labelled tZ, with or without a 500-fold excess of unlabelled tZ for the determination of non-specific and total binding, respectively (Romanov *et al.*, 2005). The apparent *K*_D_ values for [^3^H]tZ binding to different receptors were determined in saturation assays followed by data analysis in Scatchard plots. To study the ligand specificity of binding, various unlabelled cytokinins at different concentrations were added together with [^3^H]tZ. Probes were incubated on an ice bath for 40min (spheroplasts) or 60min (microsomes), then centrifuged at 16 000 *g* at 4 °C for 3min (spheroplasts) or 20min (microsomes). The supernatant was completely removed using a vacuum pump. A 200 μl aliquot of 96% (v/v) ethanol was added to the pellet and extraction was allowed to proceed for 16h at room temperature in a tightly closed tube. Extracted radioactivity was measured with a scintillation counter for 10min for each probe.

Studies of the pH influence on hormone binding were performed in a medium containing 150mM NaCl, 32mM KCl, 27mM NH_4_Cl, 2mM MgSO_4_, and 0.1mM CaCl_2_. The pH was adjusted using 50mM TRIS-HCl or MES-KOH buffers, respectively, prepared with the same medium. For pH control inside microsomes, the fluorescent dye pyranine (8-hydroxypyrene-1,3,6-trisulphonic acid) was used (Clement and Gould, 1981).

### Homology modelling of protein structures

Amino acid sequences of the proteins under consideration were retrieved via Pubmed Protein (http://www.ncbi.nlm.nih.gov/protein) (access codes: AHK3, Q9C5U1.1; AHK4, AEC05505.1; and ZmHK1, NP_001104859.1). Sequence alignment was prepared with ClustalX 2.0.11 with default parameters (Larkin *et al.*, 2007); in particular, the Gonnet matrix series was used. Sequences of templates were extracted from the Protein Data Bank (PDB; Berman *et al.*, 2000) files with the help of the universal molecular modelling program suite SybylX2.1 (Tripos International, St. Louis, MO, USA), and full sequences of target proteins were used for the construction of the alignment. The ends of the target sequences which were not represented in the template structure were trimmed.

Two kinds of models were constructed. Models used for docking studies were based on the AHK4 structure in complex with tZ (PDB ID 3T4L; Hothorn *et al.*, 2011) as template with ligands and water molecules removed, then optimized in SybylX2.1 with Tripos force field (100 iterations, Powell method, no charges) (Clark *et al.*, 1989). Subunits A and B from the 3T4L structure were used as independent templates for construction of the single subunit models. Fifty models were built with Modeller 9.10 (Šali and Blundell, 1993) for each target protein, and a thorough protocol of simulated annealing optimization was applied to each model. The best models for further docking studies were selected according to the value of the DOPE scoring function (Shen and Sali, 2006) as calculated by Modeller 9.10.

The model of the ZmHK1 complex with thidiazuron (TD) was built as a dimer based on the AHK4 structure in complex with thidiazurone (PDB ID 3T4T; Hothorn *et al.*, 2011). The ligand molecules and three water molecules located in the binding site were retained and taken into account during the modelling as rigid bodies. One hundred models were built in Modeller 9.14 and optimized using a thorough simulated annealing protocol. The best model was selected according to DOPE score and transferred to SybylX2.1. All hydrogen atoms were added (water hydrogen atoms were oriented to fulfil hydrogen bonds), MMFF94 partial atomic charges (Halgren, 1996) were assigned, and 100 iterations of minimization in the MMFF94s force field were performed. Then the Sybyl staged minimization protocol was applied with 100 iterations on each stage. The resulting model was used for comparisons and rendering.

### Molecular docking

The docking study was performed using the OpenEye workflow. The structures of the compounds were drawn in InstantJChem 5.3.0 (ChemAxon, 2011, www.chemaxon.com) and converted into OEB format with OpenEye *babel* (BABEL 3.3, OpenEye Scientific Software Inc., Santa Fe, NM, USA, www.eyesopen.com). Tautomers were generated with OpenEye *tautomers* (QUACPAC 1.6.3.1, OpenEye Scientific Software Inc.), and the most reasonable ones were automatically retained. Then conformers were generated for each molecule with OpenEye *omega2* (OMEGA 2.5.1.4, OpenEye Scientific Software Inc.; Hawkins *et al.*, 2010) (exhaustive generation, 1.0 Å r.m.s. tolerance). MMFF charges (Halgren, 1996) were assigned to all ligands (QUACPAC 1.6.3.1). Water molecules #9, 23, and 122 were kept during model preparation before docking. Molecular docking was performed with FRED 3.0.1 (OEDOCKING 3.0.1, OpenEye Scientific Software Inc.; McGann, 2011), and the results were scored with the Chemgauss4 function (McGann *et al.*, 2003). One hundred alternative poses were retained for the docked molecules. Visual analysis of the poses was performed with VIDA 4.2.1 (OpenEye Scientific Software Inc.).

### Mathematical and statistical methods

Apparent affinity constants were determined on the basis of ligand saturation and competition assays using Scatchard plots and the Pharmacology option of the SigmaPlot program (Systat Software Inc., USA). Each experiment with biological probes was repeated once or twice, and mean values and standard deviations (SDs) were calculated on the basis of Student’s criteria (*t*-test statistical program).

## Results

**Fig. 1.**
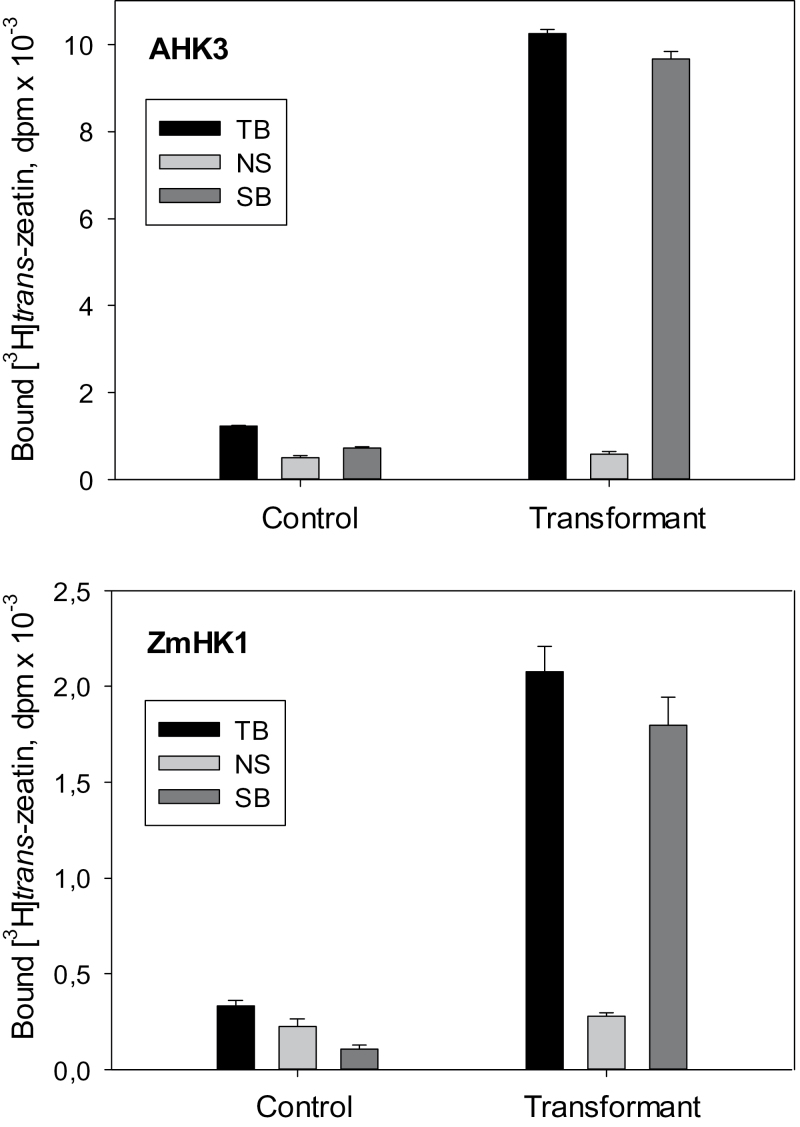
Increase in *trans*-zeatin-specific binding to plant membranes upon transient expression of cytokinin receptors in tobacco leaves. Microsomes from control and transiently transformed (‘Transformant’) plants are shown. TB, NS, and SB indicate total, non-specific, and specific binding, respectively.

Cytokinin receptors are located in membranes and cause specific binding of cytokinins to microsomes isolated from plants (Lomin *et al.*, 2011; Wulfetange *et al.*, 2011). To test whether this ability could be used to study cytokinin receptor properties systematically in a plant assay system, the cytokinin-binding activity of membranes isolated from leaves of tobacco plants (*N. benthamiana*) which had been infected or not with an *Agrobacterium* strain harboring a receptor expression cassette in its T-DNA was compared. The expression of the receptors was performed under fluorescence control. Membranes isolated from leaves of control *N. benthamiana* plants had only low cytokinin ([^3^H]tZ) binding activity, which was increased up to 20 times upon transient expression of the cytokinin receptor *AHK* genes from *Arabidopsis* or *ZmHK1* from maize (Fig. 1). This rise in activity corresponded mainly to specific binding, testifying the high affinity of the interaction. More than 95% of [^3^H]tZ-specific binding was due to the foreign receptors. This large increase in microsome binding activity over the background value allowed the properties of each individual receptor to be studied upon its transient expression.

### Cytokinin binding dependence on media conditions

Similarly to the bacterial test system (Romanov *et al.*, 2006), the specific binding of labelled tZ to the different receptors appeared to be quite rapid, reaching a plateau in 20–40min (Supplementary Fig. S1 available at *JXB* online). Temperature had an ambiguous effect on binding. AHK3 showed a significant (20–30%) increase in hormone binding at 23 °C compared with 0 °C (ice bath). This was in contrast to the bacterial assay where the highest level of hormone binding to AHK3 was observed at 0 °C (Romanov *et al.*, 2006). ZmHK1 did not show reproducible changes in hormone binding under these temperature conditions (Supplementary Fig. S1). All temperature-dependent shifts resulted from changes in tZ-specific binding while non-specific binding was not affected by temperature and remained at a low and constant level.

The influence of pH on the ability of the receptors AHK3, AHK4, and ZmHK1 to bind labelled tZ was investigated between pH 5 and pH 9.5. MES and TRIS were used to buffer the pH intervals 5–7 and 7–9.5, respectively. The identity of pH values in the medium and inside the membrane vesicles was confirmed using the pH-sensitive fluorescent dye pyranine (Supplementary Table S1 at *JXB* online).

**Fig. 2.**
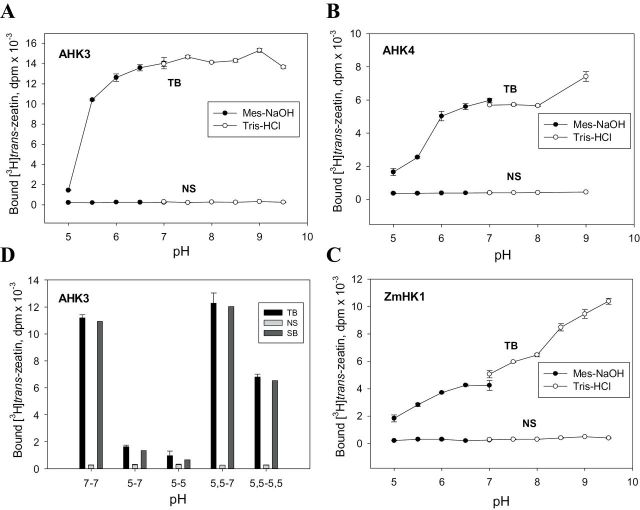
pH dependence and reversibility of cytokinin–receptor interaction. Binding of *trans*-zeatin to AHK3 (A), AHK4 (B), and ZmHK1 (C) in the plant assay system at different pH values. Reversibility of ligand binding to AHK3 following a pH shift is shown in (D); initial and final pH values are indicated by numbers below the abscissa. TB, NS, and SB indicate total, non-specific, and specific binding, respectively.

The pH dependence of [^3^H]tZ binding to receptors is shown in Fig. 2A–C. At pH 5, the hormone-specific binding of AHK3 was negligible (Fig. 2A) while AHK4 and ZmHK1 demonstrated low but noticeable binding (Fig. 2B, C). With increasing pH, a rise of hormone binding by all AHK receptors reaching a plateau at pH ~6.5–7 was observed. Addition of the channel-forming peptide alamethicin (10 μM) did not change the shape of the curve (except for a small change at the highest pH; Supplementary Fig. S2 at *JXB* online). This proves the genuineness of the measured pH dependence inside vesicles.

It was tested whether the pH influence on the hormone–receptor interaction is reversible. To this end, a part of the microsome samples was transferred after incubation at low pH to a medium with pH 7 and the level of hormone-specific binding was determined. The data showed that the decrease in the binding at pH 5.5 was fully reversible when membranes containing AHK3 were transferred again to the pH 7 buffer (Fig. 2C). However, after incubation at pH 5, the hormone-binding ability was not restored upon transfer to optimal pH conditions (Fig. 2C). This indicates an irreversible rearrangement in the ligand-binding domain of AHK3 caused by incubation at pH 5.

The pH dependence of tZ-specific binding to ZmHK1 was markedly different from the binding to AHK receptors (Fig. 2D). All receptors showed an apparent minimum of specific binding at pH 5. However, with increasing pH, a steady rise in cytokinin binding to ZmHK1 was observed, reaching a maximum at pH 9.5. In contrast, such an alkaline pH range had little influence on ligand binding to the AHK receptors. Non-specific binding in all cases was pH independent and close to zero.

### Ligand specificity of receptors

There are numerous cytokinin metabolites in the plant cell, and their inherent biological activity is often not clear. It is, therefore, of particular interest to determine the affinity of the receptors for different cytokinins and their derivatives. The investigation of the receptor ligand specificity was performed via a series of dose-dependent binding assays with cytokinin bases and their ribosides.

**Fig. 3.**
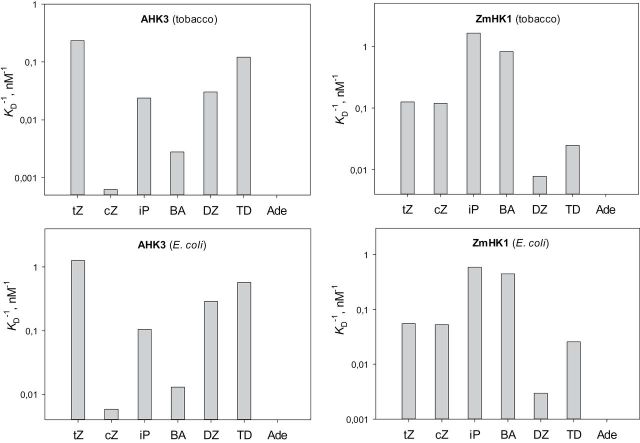
Affinity constants of various cytokinin bases for AHK3 and ZmHK1 receptors in different assay systems. The affinity constants were measured in a plant (tobacco microsomes) or bacterial (*E. coli* spheroplasts) assay system as indicated. The ordinate (log scale) shows the apparent *K*_A_=*K*_D_^–1^ which positively correlates with the ligand affinity of the receptor. tZ, *trans*-zeatin; cZ, *cis*-zeatin; iP, isopentenyladenine; BA, *N* ^6^-benzyladenine; DZ, dihydrozeatin; TD, thidiazuron; Ade, adenine.

The apparent affinity constants (*K*_A_, association constant=1/*K*_D_, dissociation constant) of the hormone–receptor complexes were determined on the basis of saturation assays with [^3^H]tZ, and competition assays with various doses of unlabelled cytokinins. Comparison of AHK3 and ZmHK1 showed (Fig. 3) that the receptors differed greatly in absolute and relative affinities for cytokinins, in accordance with previously published data (Spichal *et al.*, 2004; Romanov *et al.*, 2006; Romanov and Lomin, 2009; Lomin *et al.*, 2011). Among the natural cytokinin bases, tZ had the highest affinity for AHK3 (*K*_A_=0.23nM^–1^), followed by dihydrozeatin (DZ) and iP, while BA and especially cZ showed the lowest affinity. The affinity of AHK3 for tZ was 375 times stronger as compared with the affinity for cZ. Adenine had almost no ability to compete with tZ for binding to the receptor (Fig. 3).

As regards ZmHK1, it had high affinity for many cytokinin bases, except TD and DZ. iP (*K*_A_=1.64nM^–1^) and BA (*K*_A_=0.82nM^–1^) showed the highest affinity for this receptor in the plant system, followed by tZ and cZ with *K*_A_ values similar to each other, while TD and DZ had the weakest affinity (Fig. 3). As in the case of AHK3, adenine hardly interacted with the receptor ZmHK1.

The ligand specificity of these cytokinin receptors was assessed in parallel in the heterologous assay system using *E. coli* spheroplasts. The comparison of individual receptor properties in the two different assay systems showed that each receptor retains a typical profile of ligand specificity towards cytokinin bases (Fig. 3). Although the absolute values of affinity constants varied, their overall patterns were very similar in both test systems, so the ligand specificity profiles of individual receptors were stably reproduced irrespective the assay system used.

### Interaction of receptors with cytokinin ribosides

**Fig. 4.**
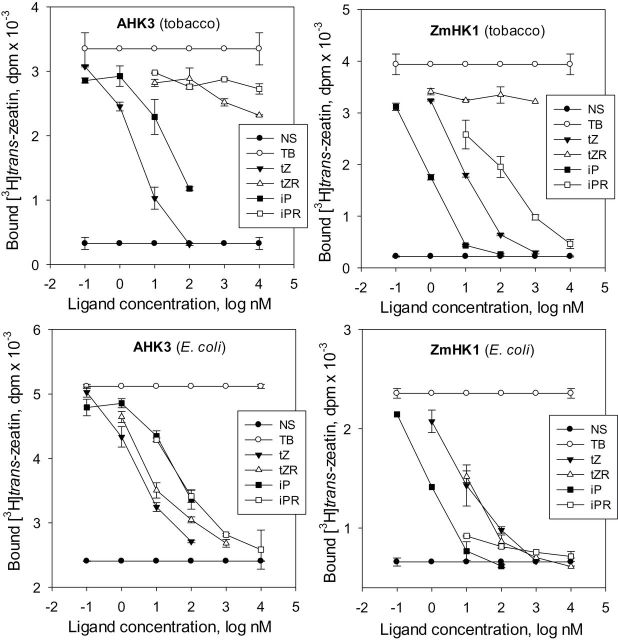
Interaction of cytokinin ribosides and bases with receptors AHK3 and ZmHK1. Receptor–ligand interaction was measured in a plant (tobacco microsomes) or bacterial (*E. coli* spheroplasts) assay system as indicated. tZ, *trans*-zeatin; tZR, *trans*-zeatin riboside; iP, isopentenyladenine; iPR, isopentenyladenosine; NS and TB indicate non-specific and total binding, respectively.

Of special interest is the interaction of the receptors with cytokinin ribosides since data available in the literature are contradictory (Heyl *et al.*, 2012). The affinity of two ubiquitous natural ribosides, *trans*-zeatin riboside (tZR) and isopentenyladenosine (iPR), for the receptors was tested and compared with that of the corresponding bases (Fig. 4). Competitive experiments with *E. coli* spheroplasts confirmed previous data (Romanov *et al.*, 2005, 2006; Romanov and Lomin, 2009; Stolz *et al.*, 2011): ribosides were able to displace labelled tZ from the hormone–receptor complex quite effectively and similarly to the corresponding cytokinin bases. However, in the plant assay system, ribosides behaved in a different way (Fig. 4): in most cases, the ribosides hardly competed with labelled tZ for binding to the receptors. The only exception was the ZmHK1–iPR interaction, although the affinity of the receptor for the ligand was at least two orders of magnitude lower than in the case of the ZmHK1–iP interaction. Supplemental assays with AHK4 corroborated the inability of cytokinin receptors to bind *N* ^9^-ribosylated cytokinins with high affinity (Supplementary Fig. S3 at *JXB* online). Thus, in the case of cytokinin ribosides, the results of binding assays strongly depend on the test system.

Additionally the isolated *E. coli* membranes containing the cytokinin receptors were checked for their ability to interact with cytokinin ribosides. Experiments demonstrated that cytokinin ribosides strongly interacted with the receptors within the isolated bacterial membranes, similarly to the interaction of ribosides with spheroplasts (Supplementary Fig. S4 at *JXB* online). Thus, the homologous assay system based on the isolated plant membranes has unique properties and cannot be replaced by isolated bacterial membranes (at least from *E. coli*).

### Investigation of the full-length receptor AHK2

**Fig. 5.**
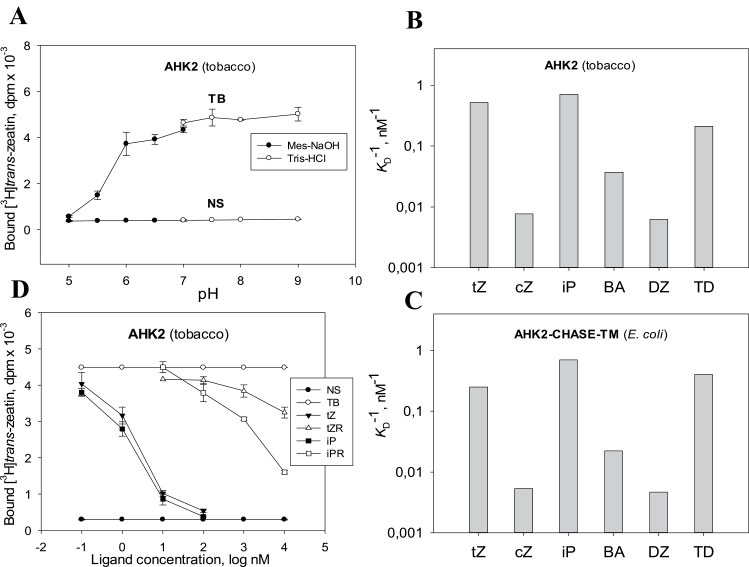
Ligand binding properties of full-length receptor AHK2 assayed in the plant assay system. Typical pH dependence of ligand binding is demonstrated in (A). Affinity constants for various cytokinin bases (B) are shown in comparison with constants of the same ligands for the AHK2 sensor module (AHK2–CHASE–TM) (C) studied in a bacterial (*E. coli*) assay system (Stolz *et al.*, 2011). In contrast to free bases, cytokinin ribosides hardly bind to the receptor (D). For abbreviations, see legends to Figs 3 and 4.

Another advantage of the plant test system is the possibility to study full-length receptors which are hardly expressed in bacteria. For example, the expression of AHK2 in *E. coli* met with difficulties presumably due to toxicity of this protein for the bacteria. As a consequence, only the separated sensor module of AHK2 has been studied so far in a heterologous binding assay system (Stolz *et al.*, 2011). However, the same receptor was successfully expressed in tobacco leaves after *Agrobacterium*-mediated transformation with a DNA construct harbouring the full-length *AHK2* gene (Wulfetange *et al.*, 2011). To analyse the ligand specificity of full-length AHK2, membranes were isolated from tobacco leaves expressing this receptor under fluorescence control. The expression of AHK2 resulted in a strong rise of the cytokinin-binding ability of the isolated membranes similarly to other expressed cytokinin receptors. This made it possible to investigate for the first time the ligand-binding properties of full-length AHK2 (Fig. 5). The pH dependence of cytokinin binding by AHK2 closely resembled that of AHK3, with nearly zero binding at pH 5 (Fig. 5A). Again, cytokinin bases strongly interacted with the receptor (Fig. 5B) whereas cytokinin ribosides did not (Fig. 5C). The ligand specificity profile of full-length AHK2 (Fig. 5B) appeared to be very similar to the profile of its sensor module (Stolz *et al.*, 2011; Fig. 5D) with only minor deviations.

The apparent affinity constants and profiles of ligand preference for individual cytokinin receptors studied in the plant assay system are presented in Table 1. In general, all studied receptors (including AHK4) displayed high and similar affinities for tZ with a *K*_D_ within the range of 2–8nM, but the affinities for other cytokinins varied greatly. This concerned primarily cZ, iP, and BA which differed in their affinities for the receptors sometimes by up to 200- to 300-fold.

**Table 1.** Apparent K_D_ *(nM ±SD*) of various cytokinin–receptor complexes tested in the plant assay systemData correspond to assays at pH 7.4 and 0–4 °C

Cytokinin base	AHK2	AHK3	ZmHK1
*trans*-Zeatin (tZ)	1.93±0.26	4.26±1.69	7.97±1.96
*cis*-Zeatin (cZ)	130±18	1602±326	8.39±2.58
Isopentenyladenine (iP)	1.42±0.52	42.0±13.5	0.61±0.27
Benzyladenine (BA)	26.9±9.9	359±10	1.22±0.56
Dihydrozeatin (DZ)	161±55	33.1±6.2	128±47
Thidiazuron (TD)	4.76±2.05	8.23±1.20	40.1±12.9
Adenine	ND	>10 000	>10 000
Activity ranking	iP≥tZ>TD>BA>cZ≥DZ	tZ>TD>DZ≥iP>BA>cZ	iP≥BA>tZ≈cZ>TD>DZ

ND, not determined.

### Molecular modelling

**Fig. 6.**
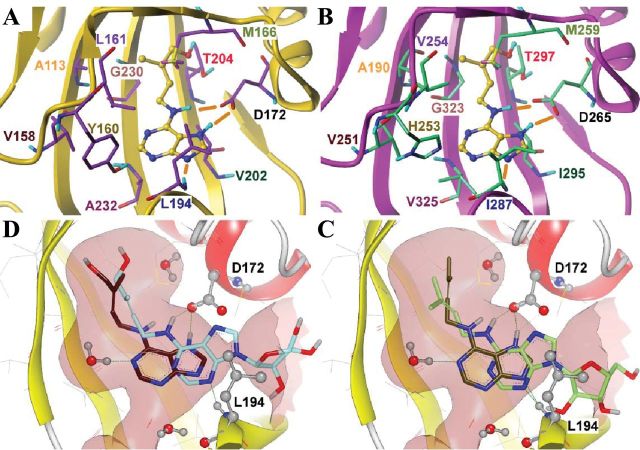
Molecular modelling of cytokinin–receptor interaction. (A, B) Models for cytokinin (tZ) binding, predicted by molecular docking, to receptors ZmHK1 (A) and AHK3 (B). The ligand is rendered in a ball-and-stick representation with yellow carbon atoms; residues of the receptors forming the hormone-binding site surface are shown as sticks; the backbone is shown in cartoon representation. Residue numbers are coloured to indicate similar positions in the different receptors. Nitrogen atoms are coloured in blue, oxygen in red, hydrogen in cyan, and sulphur in light yellow. Hydrogen bonds are shown as orange sticks. Non-polar hydrogen atoms and water molecules are omitted for clarity. Both A and B have been produced using SybylX2.1. (C, D) Comparison of predicted binding modes of cytokinin bases and cytokinin ribosides, iP versus iPR (C) and tZ versus tZR (D), to ZmHK1. Hydrogen bonds are shown as green dashed lines. Important hydrogen-bonding residues and water molecules are shown in ball-and-stick representation. Conserved bond-forming residues (D172 and L194) in the binding cavity are indicated. The surface of the binding cavity is coloured rose. Both C and D have been produced using VIDA 4.2.1.

To gain further insight into the cytokinin–receptor interaction, the structures of the ligand-binding sensor modules of the cytokinin receptors from *Arabidopsis thaliana* and *Zea mays* were modelled by homology using the AHK4 sensor module structure as a template (Hothorn *et al.*, 2011). The high level of sequence identity between the templates and models made modelling a robust and straightforward procedure. The overall structures of the obtained models closely resemble the structure of the template, and hormone-binding sites are generally conserved (Fig. 6A, B). The highly conserved aspartate residues forming two hydrogen bonds with cytokinins play a crucial role in ligand binding. Certain amino acid variations in the binding pockets may be responsible for hormone selectivity profiles.

Docking studies were performed for tZ and iP and for the corresponding ribosides, tZR and iPR (Fig. 6C, D). Free hormones are scored much more favourably compared with their riboside counterparts (scores are given in Supplementary Table S2 at *JXB* online), thus supporting the experimental data. Docked poses of tZ and iP in the hormone-binding sites in the PAS domains of the studied receptors (Fig. 6C, D) are similar to the poses observed in the X-ray structures of AHK4 complexes with the corresponding molecules (Fig. 6A, B; Hothorn *et al.*, 2011). On the other hand, ribosides cannot form the same hydrogen-bonding patterns and the same orientations in the binding site: top scored poses for cytokinin ribosides suggest riboside moiety binding in the cavity occupied by the cytokinin aliphatic tail (not shown). In addition, several lower scored poses similar to the poses of free cytokinins were generated, but the hydrogen-bonding pattern as exemplified by the ZmHK1-binding site is still not the same (Fig. 6C, D). Due to the presence of the riboside moiety in position *N* ^9^ of adenine, a hydrogen bond donor cannot emerge in position *N* ^7^, thus exchanging a hydrogen bond to Asp172 present in the free bases with unfavourable *N*···*O* interaction. The hydrogen bonding of cytokinin ribosides with Leu194 is realized through *N* ^3^ instead of *N* ^9^, leading to the elimination of a favourable interaction with a water molecule. The most important factor leading to general loss of affinity of ribosides is very likely the steric bump between the riboside moiety and the loop β6–β7 (shown in Supplementary Fig. S5 at *JXB* online) containing Leu194 and covering the binding site. Sensor module modelling of AHK2 and AHK4 showed similar features of these receptors (not shown). In the case of AHK3, possessing a smaller cytokinin-binding cavity due to the presence of bulkier residues (Fig. 6B), no poses of ribosides are generated inside the cavity at all. The larger cavity volume of PAS domains of the other receptors might explain their ability to bind iPR, though with low affinity.

## Discussion

Proteins within cell membranes interact with lipid molecules, with other proteins, as well as with each other. These interactions influence the activity of the membrane proteins (Lee, 2003, 2004). Therefore, it made sense to investigate the functioning of membrane proteins in an environment as close as possible to the natural one. Cytokinin receptors are typical transmembrane enzymes whose sensor and catalytic domains are mandatorily located at the opposite sides of the membrane (Steklov *et al.*, 2013). Previous assay systems used for the investigation of ligand-binding properties of the cytokinin receptors were mainly based on transformed *E. coli* (Yamada *et al.*, 2001; Spichal *et al.*, 2004; Yonekura-Sakakibara *et al.*, 2004; Romanov *et al.*, 2005, 2006; Romanov and Lomin, 2009). Later, in order to improve the accessibility of the receptors for ligands, bacterial spheroplasts (i.e. bacteria lacking the outer envelope) were employed (Lomin *et al.*, 2011; this study). However, the question of how far the results obtained in heterologous assay systems reflect the reality in the plant cell still remained.

To overcome this problem, a plant assay system based on microsomes isolated from tobacco leaves transiently expressing cytokinin receptor genes was developed (Fig. 1). The data showed that cytokinin receptors overexpressed in tobacco leaves retained their ability to bind specific ligands with high affinity. The use of this new system was slightly more time-consuming compared with the bacterial system but it proved to be reliable and enabled a detailed study of ligand-binding properties of individual cytokinin receptors. The result conclusively proved the existence of significant differences between the receptors in their affinity for various cytokinins as well as in their profiles of ligand preference (Table 1). As a consequence, the hormonal activity of each cytokinin base will depend at least in part on the type of receptor(s) present in the cell. For example, tZ and iP were generally the most preferred ligands for the receptors, but with AHK3 iP was one order of magnitude less active than tZ. The latter was in accordance with a much weaker effect of iP compared with tZ on *Arabidopsis* mutants expressing AHK3 as the sole receptor (Stolz *et al.*, 2011). Some other cytokinins, namely BA and especially cZ, bind rather weakly to *Arabidopsis* receptors, but with ZmHK1 they show a high activity similar to iP and tZ. Thus ZmHK1 has a unique property: unlike the other receptors, it binds with equal affinity the *trans*- and *cis*-isomer of zeatin. This agrees well with an equal sensitivity of ZmHK1 for *trans*- and *cis*-zeatin and with other data from heterologous functional biotests (Mok *et al.*, 2005; Yonekura-Sakakibara *et al.*, 2004). As cZ and BA were found in maize in rather high concentrations (Veach *et al.*, 2003; Alvarez *et al.*, 2008; Vyroubalová *et al.*, 2009; Stirk *et al.*, 2012), the present data support the postulated function of these compounds as genuine cytokinins in maize.

The plant assay system made it possible to refine the absolute values of the apparent affinity constants of the natural cytokinins for the different receptors (Table 1). When compared with the heterologous assay system, the absolute values were rather close in the case of AHK2 but somewhat different in the case of the other two receptors (up to three times for ZmHK1 and up to 10 times for AHK3). Evidently, the membrane microenvironment influences in some way the ligand-binding properties of the receptors and the degree of this influence depends on the receptor structure. However, in spite of this, the ligand specificity profiles of receptors toward cytokinin bases were very similar regardless of the assay system used (Figs 3, 5; Romanov *et al.*, 2006; Lomin *et al.*, 2011), demonstrating that each cytokinin receptor possesses a quite stable ligand (cytokinin base) specificity profile that can be reproduced in various assay systems.

One important outcome of this study has been the unequivocal demonstration that cytokinin receptors located in a plant membrane bind only free cytokinin bases with high affinity but not the corresponding ribosides. Numerous bioassays had suggested that cytokinin ribosides possess their own hormonal activity, but previous receptor-binding assays and structural studies have produced conflicting results. Indeed, in all previously used binding or reporter activation bacterial assays with diverse receptors (from *Arabidopsis* or maize) cytokinin ribosides demonstrated substantial cytokinin activity and a high receptor affinity (Yamada *et al.*, 2001; Spíchal *et al.*, 2004; Yonekura-Sakakibara *et al.*, 2004; Romanov *et al.*, 2005, 2006; Stolz *et al.*, 2011; Kuderová *et al.*, 2015). These results were reproduced in the present study (Fig. 4). Moreover, isolated *E. coli* membranes containing these receptors gave similar results (Romanov *et al.*, 2005; Supplementary Fig. S4 at *JXB* online). However, when membranes from yeast transformed with the AHK4 receptor were used, iPR (unlike iP) was shown to be inactive (Yamada *et al.*, 2001). To explain the contradictory results in the two assay systems, the authors suggested that *E. coli* cells quickly convert ribosides into the corresponding bases which in turn activated the receptors. Finally, the 3D structure of the ligand-binding site of the cytokinin receptor supported the absence of hormonal activity of tZ ribosylated at the *N* ^9^ position since the riboside moiety did not fit into the binding pocket (Hothorn *et al.*, 2011). Experiments with all three receptors from *Arabidopsis* and ZmHK1 from maize in the plant assay system demonstrated (Fig. 4; Supplementary Fig. S3) that cytokinin ribosides do not bind or bind very weakly to the receptors, implying that they have no significant hormonal activity. Apparently, glycosidase activity that cleaves the ribose residue from the *N* ^9^ atom of cytokinin is weak or absent in membranes of eukaryotic cells (yeasts and plants), though it cannot be excluded that a weak binding activity of ribosides, especially iPR, in the plant assay system might be due to some traces of glycosidase activity as well.

The finding that cytokinin receptors do not recognize riboside derivatives has important consequences for our understanding and interpretation of cytokinin activity *in planta*. It seems comprehensible that ribosides as the main transport form of cytokinins (Sakakibara, 2006; Hirose *et al.*, 2008) are lacking inherent hormonal activity. Hence, after reaching the target tissue, cytokinins need to be ‘activated’ via conversion into the free base forms. So far only the formation of free cytokinin bases from its nucleotide precursor by the activity of phosphohydrolases named LONELY GUY (LOG) is known, but not their direct formation from ribosides (Kudo *et al.*, 2010). This indicates that phosphorylation of the cytokinin transport form may be required prior to formation of the active hormone. This would be relevant as cytokinin nucleotides are negatively charged non-diffusable molecules which might ‘trap’ the hormone in the cell (cytosol) prior to its eventual activation and action as a diffusible free base. A further note with practical implications is that the endo genous concentrations of ribosides are often substantially higher than those of the corresponding bases and are commonly interpreted as reflecting the cytokinin status of the tissue analysed. The finding of (almost) exclusive receptor–base interaction argues strongly for considering the concentrations of the free bases as the most important parameter.

How can the inability of the receptors to recognize ribosides be explained? Apparently, the process of hormone binding includes some kind of binding site closure by the loop β6–β7 (Supplementary Fig. S5 at *JXB* online), and this movement could be impaired by the presence of the riboside moiety. The initial state of the binding site is expected to be open. It is hypothesized that after the cytokinin molecule has entered the binding site in a correct orientation, loop movement is initiated, leading to the formation of a hydrogen bond between *N* ^9^ of the hormone and a conservative leucine residue (Leu194 in ZmHK1), thus defining the tight binding interaction. In the presence of a riboside residue, such movement does not lead to formation of a tight hydrogen bonding network or even does not occur at all, thus being the reason for low activity of ribosides. The structural similarity of the sensor module of different cytokinin receptors (Fig. 6; Steklov *et al.*, 2013) suggests a common mode of their interaction with ligands, but only further studies with additional receptors will show how universal this feature is.

One more advantage of the plant assay system is the possibility to analyse receptors whose study in bacteria is problematic, as is demonstrated here by the successful characterization of the ligand properties of full-length AHK2 (Fig. 5) thus overcoming previous difficulties (Stolz *et al.*, 2011). The ligand specificity profile of the full-length AHK2 appeared to be similar to the profile of its sensor module expressed in *E. coli*. The only evident difference concerned the synthetic cytokinin TD: it was less active than tZ in the case of the full-length receptor but more active in the case of the isolated sensor module. However, in both cases, the affinity of tZ and TD for both forms (full-length and truncated) of AHK2 was rather close. These data emphasize once again the stability of the ligand specificity profile which is a distinctive property of the cytokinin receptors persisting regardless of the assay system and even the integrity of the receptor itself. Hence the ligand preference of the receptor can serve as an individual passport distinguishing it from the close homologues.

It should be noted that the cloned receptors have been fused to fluorescent tags [green fluorescent protein (GFP)/yellow fluorescent protein (YFP)] to control the expression of these proteins *in planta*. In the authors’ opinion, the probability that these tags at the C-terminus of the bulky receptor protein affect the sensor module (CHASE domain) located at the opposite N-terminus is very low. The sensor module and the fluorescent tag are in all cases separated by a membrane layer and cannot come into direct contact. Studies with AHK2 and AHK4 showed that even lack of the whole cytoplasmic portion of the receptor including the catalytic and receiver domains had no significant influence on the ligand preference of its sensor module (Stolz *et al.*, 2011; this study). In addition, *in vivo* studies showed that fusion of GFP to either terminus did not impair the functionality of cytokinin receptors (Caesar *et al.*, 2011). Together, the available data do not support a significant influence of the C-linked reporter protein on the ligand-binding properties of cytokinin receptors which is therefore unlikely to occur.

Of special interest is the pH dependence of hormone–receptor binding, since the pH-dependent character may be indicative of the subcellular localization of the receptor. In fact, the pH dependence of cytokinin receptor binding served as a first hint of the intracellular localization of the receptors to the endomembrane system (Romanov *et al.*, 2006) which was corroborated in further experiments (Lomin *et al.*, 2011; Wulfetange *et al.*, 2011; Caesar *et al.*, 2011). Experiments with the plant assay system confirmed the general pattern of pH dependence for the cytokinin–receptor complex formation. In every case, hormone binding was decreasing with increasing acidity of the medium and minimal binding was observed at the lowest of all the tested pH values (pH 5; Figs 2, 5). Incubation of the receptor (exemplified by AHK3) at pH 5 led to irreversible inactivation of its ligand-binding capability. According to recent measurements, in endoplasmic reticulum (ER) lumens of *Arabidopsis* and tobacco cells, pH values are in the range of 7.1–7.5 (Martinière *et al.*, 2013), whereas the apoplast is normally much more acidic with a pH value of 4.5–5.5 (Felle, 2005). This indicates that inner cellular membranes located in a neutral or weakly alkaline microenvironment represent a more favourable platform for receptor functioning than the plasma membrane whose outer side contacts the acidic apoplast. On the other hand, it would be premature to exclude the presence of functioning receptors within plasma membranes since at pH 5.5 receptors showed a quite obvious ability to bind cytokinin (Figs 2, 5). Even at pH 5 some receptors (AHK4 and ZmHK1) retained a noticeable ligand-binding ability corresponding to 23.4% and 37.2% of the binding at pH 7, respectively.

The pH dependence of cytokinin binding by ZmHK1 showed an unexpected profile. Specific binding of tZ by this receptor rose almost linearly with increasing pH over a wide range from pH 5 to pH 9.5. To explain this dependence, one may hypothesize that this receptor serves not only as a hormonal sensor but also as a pH sensor. In the bacterial world, histidine kinases of the two-component systems have been known for a long time as pH sensors (Pflock *et al.*, 2004; Perez and Groisman, 2007; Prost *et al.*, 2007; Müller *et al.*, 2009). The structural basis for the pH sensitivity of chemoreceptor TlpB from *Helicobacter pylori* has been uncovered recently (PDB ID 3UB6; Sweeney *et al.*, 2012). It was found that the ligand-binding PAS domain of this chemoreceptor is responsible for the pH sensitivity. The presence of the bound ligand (urea) in the PAS domain is a prerequisite for the sensory function. The protonation of an aspartate residue in the ligand-binding site forming a double hydrogen bond with the urea molecule plays the key role for protein function. These structural features have a direct analogy with cytokinin receptors (Supplementary Fig. S5 at *JXB* online). Although the aspartate residues required for ligand binding are located in different positions in the PAS domains of the TlpB and ZmHK1 proteins, both can form tight hydrogen bonds with urea (TlpB) or urea derivatives such as TD (ZmHK1) (Supplementary Fig. S5).

The physiological function of the high sensitivity to pH of ZmHK1 is not clear yet. When the receptor is located in the ER, its sensor module predictably faces the ER lumen. It may be assumed that an excessive lumen alkalization enhances cytokinin signalling and leads to the expression of some cytokinin-dependent genes participating in pH regulation. Changes in cytoplasmic pH include, for example, alkalization in root epidermal cells at the hair initiation site (Bibikova *et al.*, 1998) and in leaf guard cells upon abscisic acid treatment (Gonugunta *et al.*, 2008). Therefore, a special mechanism is required to control pH homeostasis, and certain cytokinin receptors may be a part of such a mechanism. It is noteworthy that among the cytokinin-responsive genes are some which regulate membrane transport including ER-to-Golgi vesicle-mediated translocation (Bhargava *et al.*, 2013). A cytokinin-induced change of transmembrane transport could affect the pH level in the ER, thus providing a feedback mechanism to restore normal homeostasis. It is also not excluded that a rise of the pH value in the ER lumen activates the cytokinin-dependent intracellular multistep phosphorelay, thus serving as a hormone-like signal. Further studies are required to explore the potential role and functional relevance of some cytokinin receptors as pH sensor molecules.

## Supplementary data

Supplementary data are available at JXB online.

Figure S1. Temperature dependence and rate of [^3^H]tZ binding to cytokinin receptors in the plant assay system.

Figure S2. pH dependence of [^3^H]tZ binding to cytokinin receptor AHK3 in the plant assay system, in the presence of alamethicin.

Figure S3. Interaction of cytokinin bases and ribosides with the AHK4 receptor in the plant assay system.

Figure S4. [^3^H]tZ binding to isolated membranes of *E. coli* expressing *ZmHK1*.

Figure S5. Structure similarity of sensor modules of a cytokinin receptor (ZmHK1) and a bacterial pH sensor (TlpB).

Table S1. Control for pH values inside plant membrane vesicles using pyranine.

Table S2. Docking scores (Chemgauss4 function) for the best-scored poses of cytokinin bases and cytokinin ribosides in cytokinin receptors.

Supplementary Data
