# Supplementary material for: Plant membrane assays with cytokinin receptors underpin the unique role of free cytokinin bases as biologically active ligands
Source: J Exp Bot. 2015 Jan 21;66(7):1851–63. doi: 10.1093/jxb/eru522 (PMC4378623; doi:10.1093/jxb/eru522)
Supplement: Supplementary Data [file supp_66_7_1851__index.html]

Plant membrane assays with cytokinin receptors underpin the unique role of free cytokinin bases as biologically active ligands — Plant membrane assays with cytokinin receptors underpin the unique role of free cytokinin bases as biologically active ligands — Supplementary Data 

# Plant membrane assays with cytokinin receptors underpin the unique role of free cytokinin bases as biologically active ligands

## Supplementary Data

Data files

**Files in this Data Supplement:**

- Supplementary Data - Supplementary Data
