# Supplementary material for: Plant membrane assays with cytokinin receptors underpin the unique role of free cytokinin bases as biologically active ligands
Source: J Exp Bot. 2015 Jan 21;66(7):1851–63. doi: 10.1093/jxb/eru522 (PMC4378623; doi:10.1093/jxb/eru522)
Supplement: Supplementary Data [file supp_eru522_jexbot140699_file001.pdf]

Supplementary data

Table S1. Control for pH values inside plant membrane vesicles using pyranine.

The fluorescent dye pyranine (8-hydroxypyrene-1,3,6-trisulfonic acid) (Clement and Gould, 1981) was used as water-soluble membrane-nonpenetrating pH indicator. Pyranine was added to the homogenization buffer during the isolation of microsomal fractions from tobacco leaves, the final concentration was 200 μM. The vesicular suspension was filtered through a Sephadex G-50M column (Pharmacia Fine Chemicals, Uppsala, Sweden), mixed with pH-adjusted buffers and incubated for 10 min. The ratio of fluorescence at λ_{em}=510 nm inside microsomes illuminated with excitation light λ_{ex}=405 nm or λ_{ex}=460 nm was determined. Alamethicin (10 μM) was added as pore-forming dye in the incubation buffer in order to compare internal and external pH values.

pH	Ratio of pyranine fluorescence (λ=510 nm) after excitation at λ=405 and 460 nm	
	Without alamethicin	With alamethicin
6.5	0.42 ± 0.07	0.38 ± 0.13
7.0	0.81 ± 0.11	0.86 ± 0.05
7.5	1.27 ± 0.09	1.13 ± 0.07
8.0	1.93 ± 0.08	1.87 ± 0.03

Table S2. Docking scores (Chemgauss4 function) for the best-scored poses of cytokinin bases and cytokinin ribosides in cytokinin receptors. For the same receptor, lower values correspond to tighter binding.

Ligand	Cytokinin receptors			
	ZmHK1	AHK2	AHK3	AHK4
tZ	-13.24	-10.38	-7.91	-14.30
iP	-12.18	-9.96	-8.48	-13.51
tZR	-5.64	-5.02	-3.91	-5.53
iPR	-5.05	-5.48	-4.55	-6.84

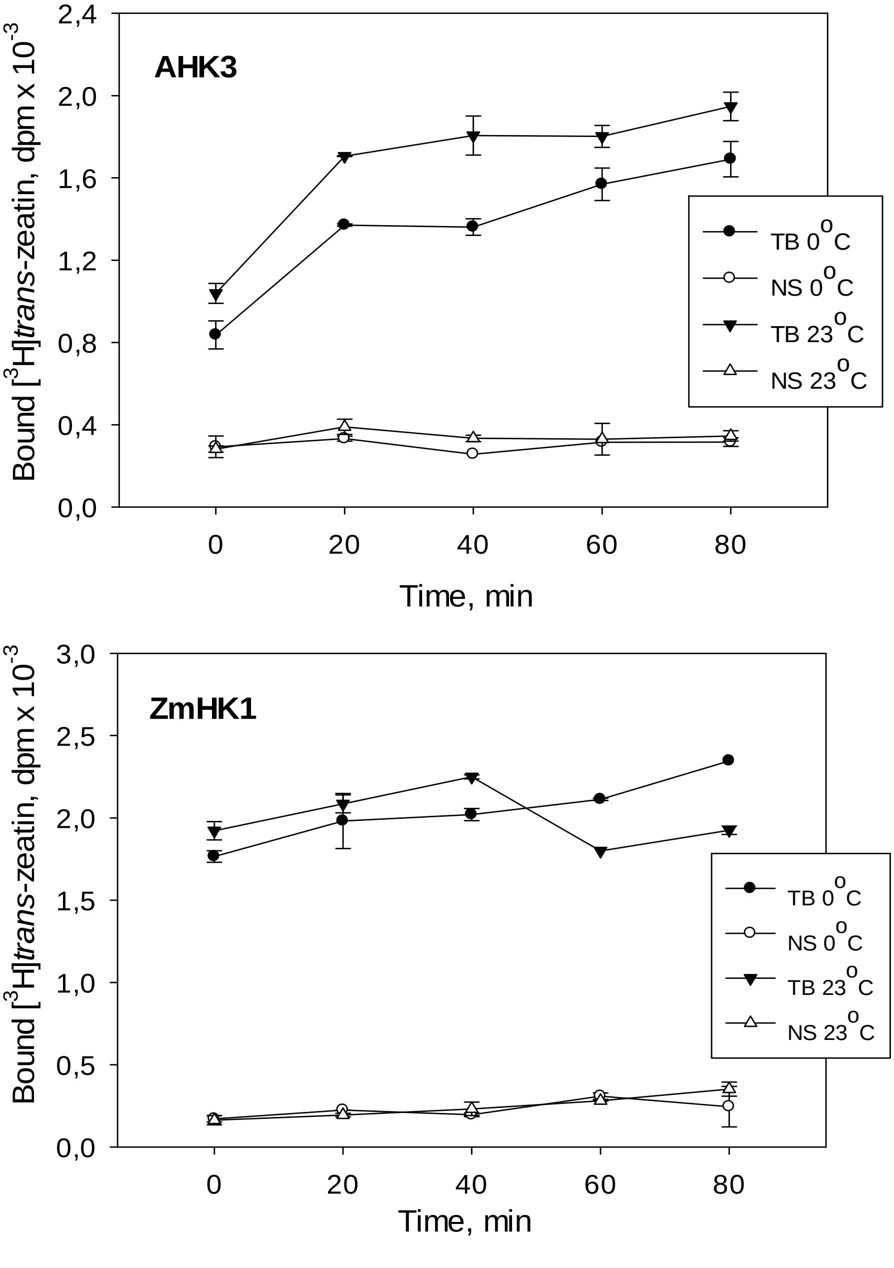

Fig. S1. Temperature dependence and rate of [^{3}H]tZ binding to cytokinin receptors in the plant assay system. TB, NS and SB mean total, nonspecific and specific binding, respectively.

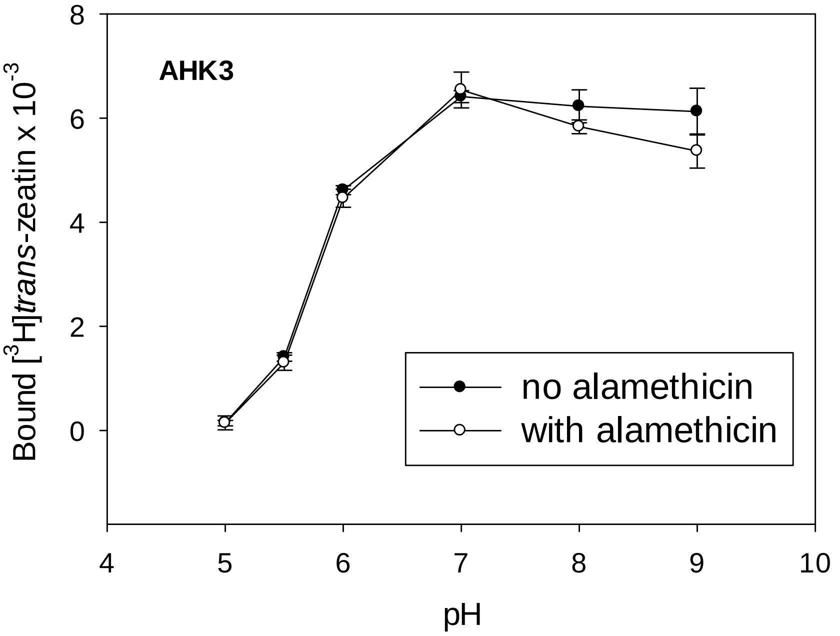

Fig. S2. pH dependence of [^{3}H]tZ binding to cytokinin receptor AHK3 in the plant assay system. The assay was performed in the absence or presence of the pore forming dye alamethicin (10 µM).

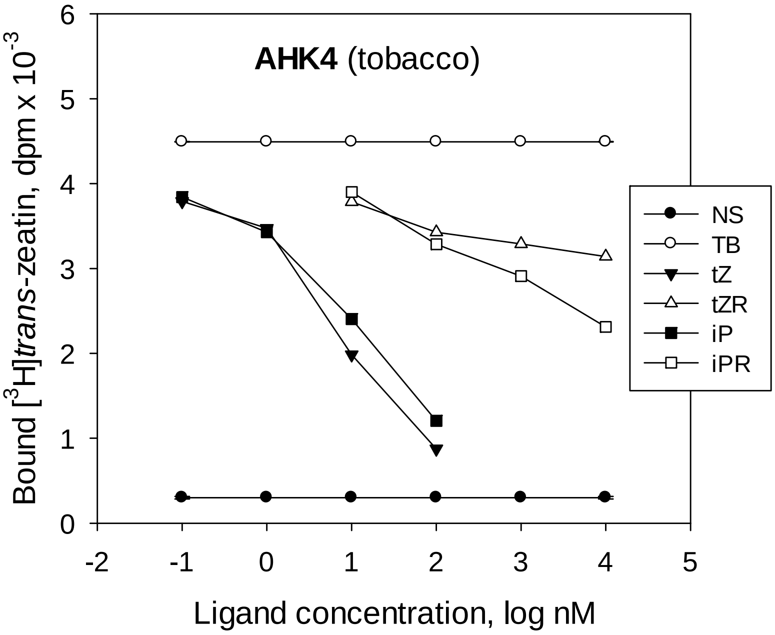

Fig. S3. Interaction of cytokinin bases and ribosides with the AHK4 receptor in the plant assay system. Abbreviations: tZ, *trans*-zeatin; tZR, *trans*-zeatin riboside; iP, isopentenyladenine; iPR, isopentenyladenosine; NS and TB mean nonspecific and total binding, respectively. The apparent K_D for tZ and iP interaction with AHK4 (pH 7.4 and 0-4 °C) were calculated to be 4.6 and 7.6 nM, respectively.

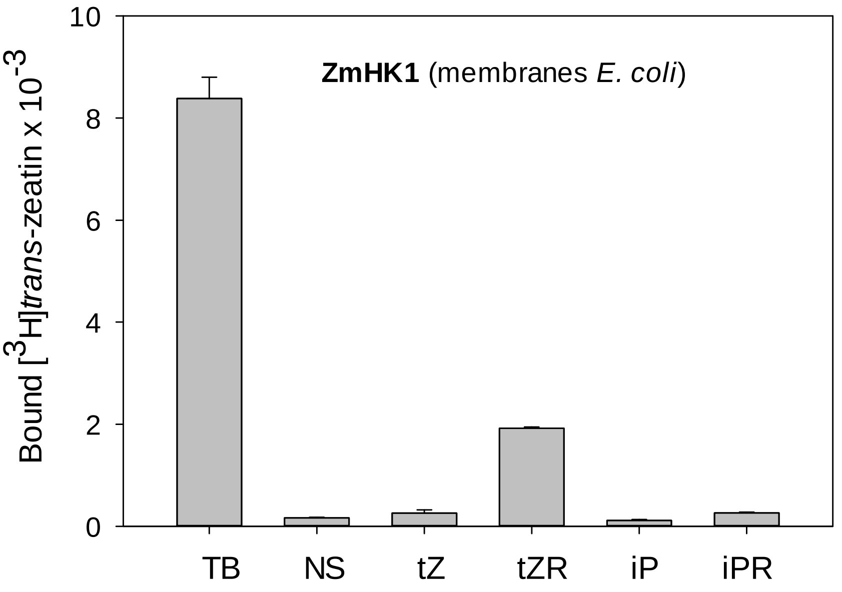

Fig. S4. [^{3}H]tZ binding to isolated membranes of *E. coli* expressing *ZmHK1*. TB and NS are total and non-specific binding, respectively. The other columns show bound [^{3}H]tZ in the presence of 1 µM unlabeled cytokinin bases or the corresponding ribosides as indicated. All tested ligands, *trans*-zeatin (tZ), *trans*-zeatin riboside (tZR), isopentenyladenine (iP) and isopentenyladenosine (iPR) strongly competed with [^{3}H]tZ binding to ZmHK1.

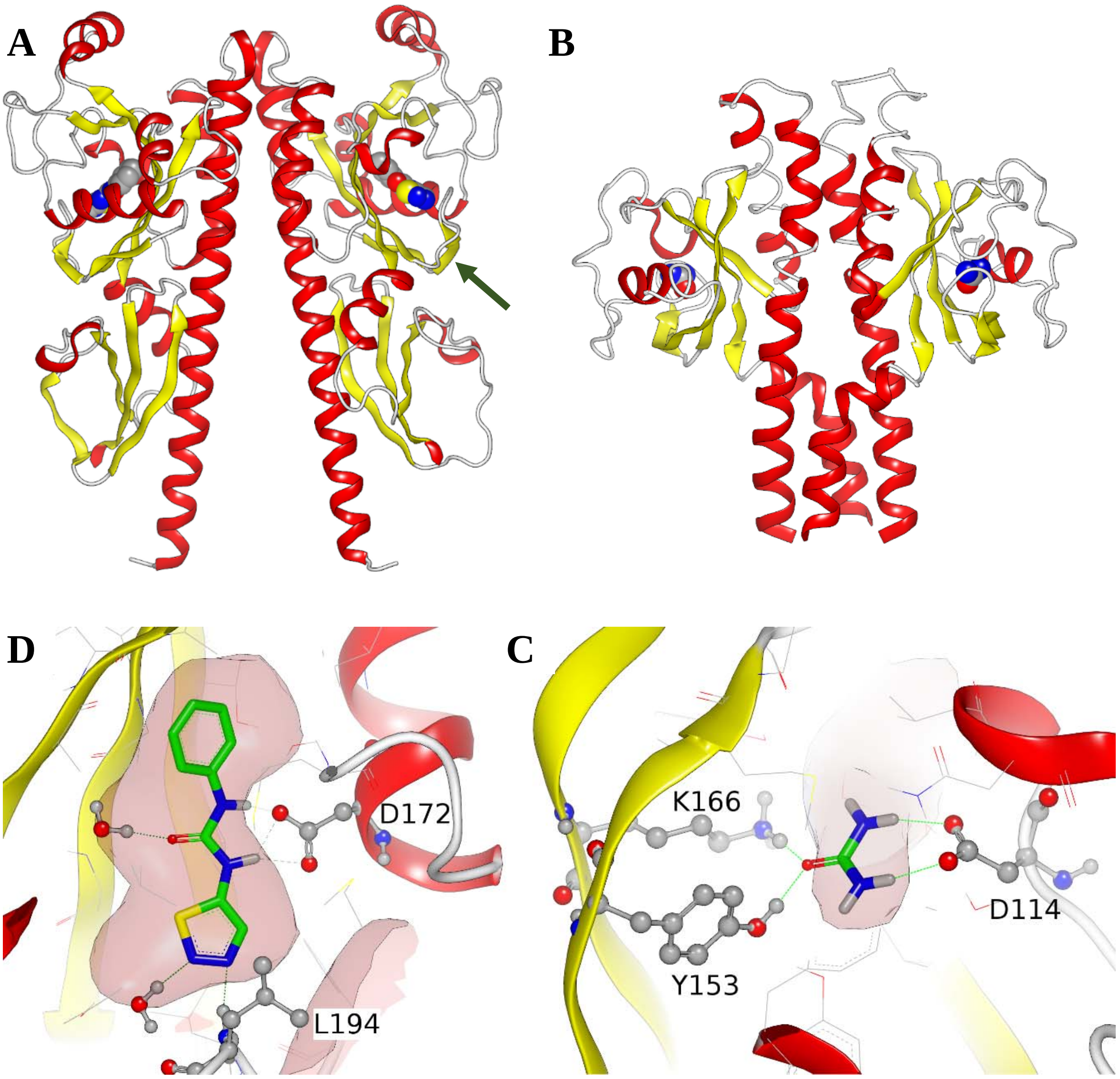

Fig. S5. Structure similarity of sensor modules of cytokinin receptor (ZmHK1) and bacterial pH-sensor (TlpB). (A, B) Overall structures of the ZmHK1 dimer complex with thidiazuron (A), and of the TlpB dimer complex with urea (PDB ID 3UB6) (B). In both cases dimerization interfaces are formed with long α-helices (in red) holding at their 3'ends ligand-binding PAS domains. Ligands are shown in space-filling representation. Arrow points to β6-β7 loop of ZmHK1. (C, D) Models for the interaction of urea with TlpB (PDB ID 3UB6, hydrogen atoms added in SybylX2.1) (C) and of thidiazuron with ZmHK1 (D). Hydrogen bonds are shown as green dashed lines. Binding cavity surfaces are colored red. The figure has been made using VIDA 4.2.1.
